# Parathyroid Hormone Secretion and Related Syndromes

**DOI:** 10.7759/cureus.30251

**Published:** 2022-10-13

**Authors:** Ketaki Dawale, Anil Agrawal

**Affiliations:** 1 Medicine, Jawaharlal Nehru Medical College, Datta Meghe Institute of Medical Sciences, Wardha, IND; 2 Pathology, Jawaharlal Nehru Medical College, Datta Meghe Institute of Medical Sciences, Wardha, IND

**Keywords:** parathyroid hormone (pth), malignancy, bones, hyperparathyroidism, hypercalcaemia, hypoparathyroidism, hypocalcaemia

## Abstract

In this article, we will get to know about the parathyroid hormone and the parathyroid gland. Its anatomy, physiology, and pathology will be delved into. There will be a brief discussion about its secretion and also about various clinical syndromes related to it. Parathormone, the parathyroid glands, regulate normal calcium and phosphorus levels in the body. An increase in the secretion of parathormone results in increased calcium uptake from the kidney, intestine, and bones, hence elevating the blood calcium level. A few mechanisms of action of this hormone are increased by the presence of vitamin D. The increase in the secretion of this hormone as compared to the normal levels is termed hyperparathyroidism. Incidence is maximum after 60 years of age. The ratio of females to males is 2:1. There are three types of hyperparathyroidism which will be described in this article. Clinical manifestations of hyperparathyroidism include skeletal disease, renal involvement, GI manifestations, psychiatric diseases, decreased neuro-muscular irritability, decreased deep tendon reflexes, muscular weakness, and atrophy. Assessment for hyperparathyroidism can be done by various diagnostic tests which are described further in this article. Medical/surgical management to cure this is also well-established nowadays.

The decrease in the secretion of this hormone as compared to normal levels is termed hypoparathyroidism. Serum calcium levels are very low, serum phosphate levels are very high, and tetany can develop. The incidence is that females are more prone than males. Assessment for acute hypoparathyroidism will show positive Chvostek sign and trousseau sign, hyperactive deep tendon reflexes, and paresthesia. Assessment of chronic hypoparathyroidism will show lethargy, weakness, fatigue, cataracts, brittle nails, dry scaly skin, personality changes, and can even cause permanent brain damage. The normal secretion process of this hormone and diseases when its secretion becomes abnormal and why that happens are briefed in this article.

## Introduction and background

Nowadays, there are various methods through which we can find out the amount of any biochemical secreted in our body. In fact, the body reacts to a particular disease so differently that it is easy to mark out the condition. Suppose the biomarkers increase in the case of heart disease, they will not increase in any other organ disease [1]. Parathyroid hormone (PTH) is a present-day treatment decision for osteoporotic postmenopausal women and hypogonadal people. As an anabolic compound that impacts bone growth, it plays an important role in osteoporosis treatment and is a proper decision to decrease the number of osteoporotic patients. Despite various clinical considerations, postmenopausal women had major vertebral and nonvertebral breaks. Regardless, a mix of PTH and bisphosphonates comes out to be a proper therapy for osteoporosis; PTH medicine taken after bisphosphonate treatment gives a fruitful outcome as monotherapy with an antiresorptive drug [2].

An endocrine disorder called hypoparathyroidism is recognized by a decrease in the amount of PTH, an 84-amino acid long polypeptide. The typical course of treatment for hypoparathyroidism involves the maintenance of standard serum calcium and phosphorus level. It decreases vertebral calcium salts and active vitamin D metabolites. Treatment will include drugs which will maintain both calcium and phosphorus levels in the serum at normal levels. Typically, hypoparathyroidism is an endocrine condition rather than a disease that can't be treated with a recombinant substance. Two recombinant PTH structures have to be combined and can be utilized to treat hypoparathyroidism [3]. There is no guarantee that hypoparathyroidism can be treated as the patient's body is unable to respond to certain drugs. Segmental bone fracture is the most common cause of the separation of bone. Autografting, which is making arrangements for colossal wounds since a small amount of bone is open for operation, can be a primary treatment for segmental bone fracture. The person's age plays an important role in whether these drugs will act or not. PTH is an anabolic hormone used in osteoporosis treatment for a bit or a while. PTH's anabolic effect on bone has given a ray of hope to newer methods of treatment [4].

## Review

Parathyroid gland

The parathyroid gland secretes the 84-amino-long peptide hormone known as PTH. It moves bone through various events of efflux of calcium from bone (unpredictable) and bone resorption (strong association) through various affiliation modes in bone tissue. It includes a potential for bone reshaping. PTH limits PTH receptors to administer bone resorption by limiting its action on bones. PTH acts on mesenchymal cells with osteoblasts, osteocytes, and T cells in association to play out an osteogenic work. It offers similar osteoclast qualities by evidently returning to osteoclast courier cells, osteoclasts, and T cells. PTH controls these Wnt (wingless-related integration site) signalling, cyclic adenosine monophosphate dependent protein kinase A pathway (cAMP/PKA), cyclic adenosine monophosphate dependent protein kinase C pathway (cAMP/PKC), receptor activator of Nf-kB ligand (RANKL)/receptor activator of Nf-kB (RANK)/osteoprotegerin (OPG), and other signalling pathways [5]. In postmenopausal osteoporotic women, the direction of parathyroid chemical teriparatide increases hip and spine bone mineral thickness and cuts down the opportunity for vertebral and nonvertebral breaks. Abaloparatide, a tantamount protein which is used as a drug, heads to the parathyroid organ, and also reduces breakage regardless of teriparatide's pharmacological side effects, strikingly in cortical bone. The blend of teriparatide (recombinant fragment of human parathyroid hormone) and the RANK-ligand inhibitor, denosumab, has been shown in preliminaries to update bone thickness and reviewed quality more than monotherapy and any routine right now available [6].

Types of hyperparathyroidism

Hypercalcemia is crucial to hyperparathyroidism. In truth, hypercalcemia can show in a broad range of clinical conditions and may not be outrageous enough to require hospitalization. This type of hypercalcemia can be unsafe and routinely causes discomfort. Severe hypercalcemia is brought about by cancer-related parathyroid-related protein or by cytokines that advance osteoclast bone deformation. Chiefly, it can be tested by evaluation lab testing, imaging, and clinical and careful associations of hypercalcemia. This should be confirmed by preliminary and differential testing when the patient is normal [7]. Table 1 shows types of hyperparathyroidism.

**Table 1:** Types of hyperparathyroidism Source: Madkhali et al., 2016 [8] (Open access)

Clinical Presentation	Calcium	PTH
Classical primary hyperparathyroidism	High	High
Primary hyperparathyroidism	High	Normal
Normocalcemic primary hyperparathyroidism	Normal	High

People affected by pseudo-hyperparathyroidism (PHP) and its related issues have surprising clinical and sub-clinical features of various sorts. People who developed this disease made the clarification that imperative illuminating, like protection from PTH, ectopic hardenings, and brachydactyly should be selected as a piece of the PHP affirmation. A sub-atomic time-to-time assessment should be done to ensure the clinical endorsement. Patients should be evaluated for specific features at the hour of the first diagnosis and amid follow-up, counting subcutaneous and more significant ectopic hardenings, neurocognitive deficiencies, PTH, thyroid stimulating hormone (TSH) anticipation, headway substance requirements, hypogonadism, skeletal reshapings, verbal achievement, weight gain, glucose requirement or sort type two diabetes mellitus, and hypertension [9].

Development of parathyroid gland

The fourth and fifth pharyngeal pockets are where the four parathyroid glands develop, slipping caudally to the focal neck. The parathyroid organs are enormously at risk for keeping up extracellular calcium and unbalanced phosphorus qualities due to the presence of PTH. Both non-parathyroid and parathyroid hypercalcemia are kinds of hypercalcemia. The first everyday issues are granulomatous hardships, risk, essential hyperparathyroidism (PHPT), and drugs. PHPT might be an issue that has proactively caused PTH to increase significantly. Let's suppose all the other things crash and burn. In that case, PHPT is the last retreat, considering a solitary liberal parathyroid adenoma (which represents 80% of cases) and multi-glandular problem, shown in around 15% of individuals. PHPT should have multi-glandular commitment, integrating unexpected parathyroid disease (1%), adenomas (5-10%), or hyperplasia of every four organs. In different cases, the issue is non-existent since PHPT isn't upheld by the individual or family [10]. The chance for ladies to get diseased with polycystic ovarian syndrome (PCOS) is because of its abnormal hormonal effect. Considering the effect of different endocrine changes on bone growth in PCOS patients is an essential requirement. Women with PCOS consistently experience issues with artificial substances such as gonadotrophin-releasing hormone (GnRH), insulin, and the leutinizing/follicle-stimulating hormone (LH/FSH) level, androgens, estrogens, growth hormone (GH), cortisol, parathyroid chemical, and calcitonin. This creates compounds which have a winding effect on the bone organization in human individuals. These are long-term complications in PCOS women suffering from osteoporosis. PCOS increases the opportunity for bone breakage in PCOS patients by weakening the bone structure [11].

Falls due to any cause result in a group of troubling outcomes, which cause mortality of people. The parathyroid organized gig is getting universal around here. Elevated PTH levels have been associated with sarcopenia, osteoporosis, and other age-related diseases. Most useful tests have focused on muscle quality, with few fixating on how it impacts change issues. This will regulate PTH and its formation, its apparent effects on muscles, and the relationship between PTH and its changes in the body [12]. The nuclear qualities and activities of parathyroid hormone receptor protein (PTHrP), both in normal physiology and in patients, at the opportunity for headway. Elevated PTHrP, which causes a common side-effect of squamous cell carcinoma, acts through parathyroid chemical receptors inside the kidney and is an issue that causes humoral hypercalcemia of malignancy (HHM). In contrast to what clinical examinations initially revealed, the tissue identification method and genuine radioimmunoassays delineate that a more remarkable collection of infirmities communicates PTHrP. It also contributes to the test of related hypercalcemia connected with a combination of etiologies, for example, wrecks metastatic to bone and haematological malignancies. PTHrP has different encoding quality, with unmistakable exons coding for up to 12 elective records and three specific length proteins, perhaps in a tissue-express way using three accessories [13]. Metabolic issues brought about by the unfortunate lack of the stimulatory G protein (Gs)/cAMP/PKA pathway that changes the development of PTH/PTHrP bring almost attributes of target organ non-responsiveness, which influences signs taken after the nonappearance of the shown substance. The subunit of the Gs or downstream effectors of an inside and somewhat obscure pathway, for example, the protein kinase A (PKA) regulatory subunit 1A and the phosphodiesterase sort 4D, could bow, happening in pseudohypoparathyroidism (PHP) and related wrecks. These issues delivered the ordinary comprehension of PHP out of date since it avoids related conditions like acrodysostosis (ACRDYS) or direct heteroplasia. Thus, a new training system and structure have been recommended that assemble these issues underneath the expression "inactivating PTH/PTHrP hailing disrupting impact" [14].

Though PTHrP plays an essential role in developmental parts, parathyroid substance plays a significant effect on the ordinary level of blood calcium and phosphate. A tantamount receptor, the PTH/PTHrP receptor, is formed by the two peptides (B G-protein-coupled receptor). The different endocrine versus paracrine/autocrine structures for control and improvement is the reason for these standard exercises' ligands. The level of clinical difficulties because of the excess requirement of one or the other peptide, as well as the demonstration that an uncommon combination of PTH forms an abnormal bone mass, causes osteoporosis, making it evident how crucial PTH and PTHrP are. Thus, there's a more apparent fascination in figuring out the parts of PTH/PTHrP receptor advancement and then looking around for more reasonable peptide or non-peptide agonists that have affectability at this receptor when used topically rather than parenterally [15]. PTHrP could be a compound that controls progression and goes about as a myorelaxant and calciotropic substance like PTH. The stream frame depicts imperative perspectives of PTHrP pharmacology and physiology, for example, (a) the evident unmistakable verification of present-day peptides and receptors of the PTH/PTHrP structure; (b) the indeed tracked down nuclear parts of PTHrP and the control of PTHrP as an intracrine regulator of cell upgrade and cell passing; (c) the physiological and developmental exercises of PTHrP inside the cardiovascular and renal glomeruli-vascular plans. These new improvements have fortified our grip on the pathophysiological guideline of PTHrP and will make it more straightforward to see the value in its extensive use in various issues [16].

There is a solicitation of clear events of PHPT due to eutopic and ectopic parathyroid adenomas, as well as a case with a syndromic kind of PHPT (unmistakable endocrine neoplasia sort 1) and an issue with familial hypocalciuric hypercalcemia (FHH) sort 1 taking into thought a CASR inactivating modify, to advance assessments and the heads of parathyroid-related hypercalcemia. For the explanation of satisfying the necessities of differential help, more outlines of normocalcaemic hyperparathyroidism and optional hyperparathyroidism are shown. While the baffling ectopic parathyroid adenomas demand more complex tests and sound methodology, for example, video-helped thoracoscopic improvement, the traditional eutopic parathyroid adenomas are genuinely dealt with parathyroidectomy. Treatment isn't imperative for the all-around less visit FHH. To avoid abnormal exchange, an examination of families with FHH is done to find individuals with this acquired disorder [17]. Brand-name gadgets, for example, bone mineral densitometry, bone histomorphometry, the examination of pointers of bone tumours, and other clinical assessments, have all followed up on the development of rules to assist with orchestrating decisions for parathyroid change or the clinical association. Chance hypercalcemia can routinely be perceived from veritable hyperparathyroidism by the presence of a specific issue. The immunoradiometric considers parathyroid texture, which dampens severe hyperparathyroidism. The upgrade thing, PTHrP, which undeniably happens in everyday hypercalcemia, might be used to choose the various clinical attributes of hypercalcemia of harmed [18].

Patients having the risk of hypercalcemia are those who have family members with the condition of a defective endocrine pancreas, thyroid, pituitary, adrenal gland, or long bone. The piece of the parathyroid chemical inside the serum should be used to perceive a run of hypercalcemias. Subsequently, standard plans are considered essential or unnecessary to a parathyroid organ tangle. The family has a history of hypercalcemia. Various other medications bring down the PTH level. Syndromic features consolidate work area fulfilment for the comprehension or their family members, parathyroid mix (either anticipated some time as of late a new improvement or guaranteed amid parathyroidectomy), striking or disturbing parathyroid malignant growths, a family background of severe hyperparathyroidism, and the beginning of severe hyperthyroidism some time as late as the age of 50 years. A study on the calcium-chasing receptor quality is the most common speculation in patients with direct hypercalcemia, an average PTH level, and relative hypocalciuria. This speculation is persistently different from focal hyperparathyroidism, especially when there's no apparent family background of hyperparathyroidism, as is routinely the situation [19].

Experiments

Indian hedgehog (Ihh), which leads its exercises through the layer receptor settled yet interatomic appropriately with hedgehog-assistant protein, is the wellspring of PTHrP. Mice which lost PTHrP show faster chondrocyte break, somewhat defective endochondral cycle bone solidifying, and vague anyway, more-unimaginable slips away than those compared to PTH 1 receptor (PTH1R)-dead animals. In transgenic mice that overexpress PTHrP and are determinedly impacted by the alpha1 (II) procollagen support, the tangled portrayal of these skeletal openings, i.e., a senseless deferral in chondrocyte pack and endochondral setting, is seen. Besides, two natural circumstances in people seemed to have outlandish characteristics in chondrocyte increment and assembly, which are unquestionably honed by changes inside the PTH1R [20]. Regardless of how they are phenomenal in rodents, Syrian hamsters, and canines and evident in mice, parathyroid organ neoplasms are extraordinary over a broad run of evaluation focus and neighbouring species. Adenomas, carcinomas, and expansive and restricted hyperplasia of the parathyroid organ are dealt with. The defilements could be significant or ineffective. Essential parathyroid tissue is feeble to trophic ruin, including practical afflictions. Humoral hypercalcemia of harmed could be a confusion portrayed by hypercalcemia, hypophosphatemia, and broadened osteoclastic bone resorption that impacts both human and animal patients with unquestionable reverting neoplasms. Cells make parathyroid substance-like parts, which bind to PTH receptors inside the kidney and bone and cause the clinical indications of HHM [21].

PTH secretion and regulation

**Figure 1:**
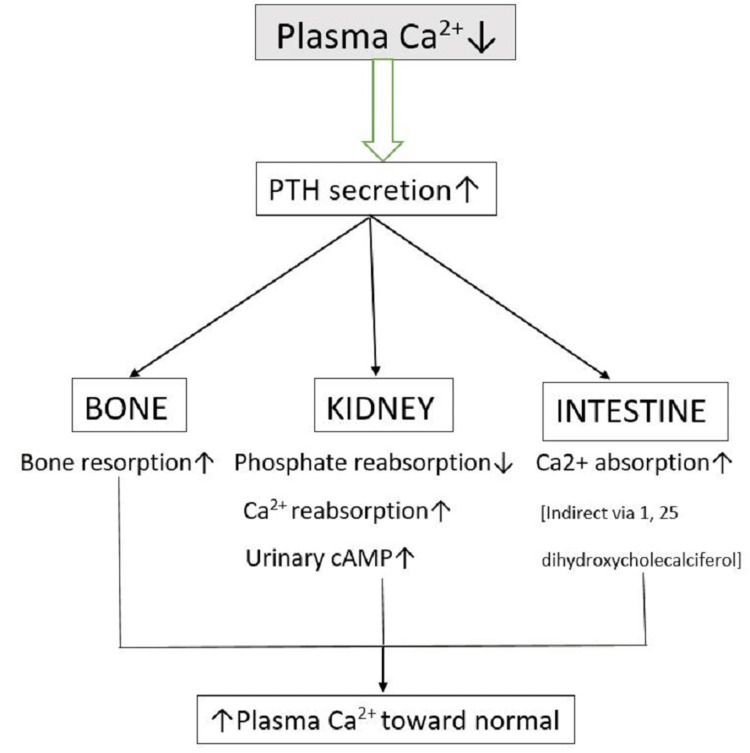
PTH secretion and regulation PTH = Parathyroid Hormone; Ca2+ = Calcium; cAMP = Cyclic Adenosine Mono Phosphate Image credit: Author Ketaki Dawale

PTH, a substance conveyed by the parathyroid gland, basically influences blood calcium levels. It incites renal 1-hydroxylase and the firm edge of vitamin D mix (1,25[OH]2D3). PTH directly impacts these battles to animate calcium release from the bone and calcium resorption. Pointers of optional hyperparathyroidism related to chronic kidney disease (CKD) consolidate an augmentation in serum fibroblast growth factor-23 (FGF23), a rot in renal 1,25[OH]2D3 mixture, and a looking at a drop in its serum levels, a deterioration in gastrointestinal calcium homeostasis, and, in a short time later stages, hyperphosphatemia and lifted PTH levels. PTH causes poorly designed effects and indications of the uremic condition, counting bone issues, skin and fragile tissue calcification, cardiomyopathy, immunodeficiency, a need for erythropoiesis, an addition in imperativeness use, and an expectation for muscle [22]. Related to pituitary adenylate cyclase (AC)-beginning polypeptide and vasoactive gastrointestinal peptide (VIP), the VIP-secretin-glucagon group of peptides additionally integrates exendins and headway substance-conveying compound. Secretin, glucagon, gastric inhibitory polypeptide (GIP), and parathyroid chemical are substances of this accumulation. Everyone in this peptide family has a surprising homology of amino-horrendous progression and binds to G-protein-coupled receptors, whose principal pathway generally integrates the phospholipase C/protein kinase C and A. Other than people of the VIP-secretin-glucagon family, others are not that essential. The pituitary gland again plays a vital role in maintaining parathyroid hormone [23]. Figure 1 shows PTH secretion and regulation.

PHPT might be a typical endocrine condition that impacts the parathyroid organs because of adenomas (80-85%), hyperplasias (10-15%), or carcinomas (1%). Atomic-based assessments of heritable P-HPT issues have given head information into the data on separating parathyroid hyperplasias and movement. This evaluation will underline how we might retouch these injuries at the sub-atomic level and focus on the essentials for social affair parathyroid proliferative worries. Regarding current PTH treatment methodologies, radiological imaging strategies are used to assess intraoperative parathyroid substances [24]. Focal hyperparathyroidism could be a typical endocrine disorder that happens because of hypercalcemia growing by the parathyroid chemical spread. Generally, it happens eccentrically 95% of the time; sometimes, it is enormous for domestic issues. The nonheritable current turn of events often shows at a more energetic age than these crashes, which likewise occur in bafflingly exact totals inside the two sexual presentations. The differential finding is reliably genuinely organized, yet it is pivotal to how patients and their friends and family associate with each other. The certified precision has been dissolved by the straightforwardness of unambiguous hereditary testing, allowing early location in asymptomatic relatives. Before genetic testing, a definitive determination must be accomplished in compelling circumstances considering clinical realities and familial genealogy [25]. Non-surgical hypoparathyroidism, known as idiopathic hypoparathyroidism, might be gathered into three elementary classes: issues with parathyroid improvement, issues with parathyroid compel, and severe pain to the parathyroids. Enormous hypoparathyroidism can be brought about by the inconvenient effects of magnesium [26].

In parathyroid contamination patients and those without it, raised blood parathyroid chemical is connected with an extended likelihood of cardiovascular passing, counting unexpected cardiac arrhythmias. In little evaluations, PTH levels have been associated with changes in heart conduction and repolarization and believed PTH's effect on serum calcium interference with changes inside the QTc or the QT stretch. To be sure, disregarding the way that there's little research associating PTH with changes in electrical conduction and repolarization liberated from serum calcium, some are demonstrating that PTH might affect cardiovascular physiology without affecting serum calcium [27].

PTH malignancy

To this, women with tall stature had a speedier pace of dysplasia in adenomas of the distal colon. Maintenance of blood vitamin D, age, plasma creatinine, BMI, diabetes, and hepatic steatosis, continued to be areas of strength for women. Serum PTH fixations were not associated with male colorectal injuries. These data recommend that extended PTH serum fixations might affect the redesign of pernicious colorectal improvement, as shown by different significant paces of adenomas, verifiably with dysplasia, in females. More is expected about what PTH means for colon carcinogenesis and how it connects with the sex [28]. Levels of progression in either bone resorption or renal barrel-shaped calcium reabsorption can prevail as the security for extended calcemia, contingent upon the reason. In drawn-out serum, lots of parathyroid glands were created, and there is a difference in the renal system that changed calcium reabsorption resulting in hypercalcemia. The ectopic difference in one particular parathyroid substance has been delineated on fair two or three occasions [29].

As various as 10% of hospitalized patients who require change might be impacted by hypocalcemia. We saw 12 prominently possible bits of probability for hypocalcemia. The aetiology may constantly be chosen at the bedside, considering the serum parathyroid compound levels, creatinine, phosphate, magnesium, creatine kinase, liver proteins, and 25(OH)D [30]. Severe hypercalcemia was seen and depicted by levels of movement showing the Parathyroid hormone-related peptide (PTHRP). There is a strong likeness between PTH and PTHRP. Inside the bioactive amino-terminal region and its ability to support the G-protein-related seven transmembrane, PTH/PTHRP receptor are the reasons for these hypercalcemic influences. Nevertheless, considering that PTHRP has been found in a couple of fetal and grown-up tissues, it might have a collection of physiological limits, for example, the possibility of having an imperative impact on cell improvement and division [31]. Children with hypercalcemia experience firsts. In adults, hyperparathyroidism is most routinely the underlying driver. Whatever the reason, the aetiologies in kids are fascinating, maturely imparted, and various have significant genetic explanations. A basic package of the time, the figured-out condition known as hypercalcemia comes to fruition in end-organ injury. An essential part of the affiliation pathway is immediately spreading the proper finding to supply the most incredible possible consideration [32]. When contrasted with ICU control patients and good controls, patients with sepsis had out and out lower levels of 25(OH)D3 (10.53 11.3 g/l against 16.46 12.58 g/l versus 24.04 12.07 g/l); the differentiation was a lot greater for 1,25(O.H.)2D3. When looked at among changed parties, serum levels of PTH and cathelicidin in patients with sepsis were dependably out of the association [33].

Cell isolates and the treated media were chromatographed using an exchanged stage high-performance liquid chromatography (HPLC) and discarded using unambiguous PTHrP immunoassays. Each of the six cell lines included different amino-terminal PTHrP species as well as elective mid-region PTHrP animal sorts, as we had at this point seen in SKRC-1 (renal cell carcinoma) and RIN (1-141) cells [34]. Hypercalcemia can occur as a difficulty of haematological diseases associated with solid updates with bone metastases and reliable updates with scarcely any bone metastases. The last issue has a couple of similarities to focal hyperparathyroidism and is sometimes implied as HMM [35]. Regardless of how it has been outlined that two-point species might convey parathyroid chemicals, the physiological reason for this peptide's root-in point is still up. There's a strong study that the parathyroid organ's development and improvement in vertebrates are impacted by various attributes [36]. Inside the ICU, hypocalcemia is normal and fills in as an indication of a crippling perspective. The calcium levels integrate calcium extravasation, long chelation, intracellular calcium over-trouble, and adjusted PTH discharge [37].

## Conclusions

PTH is an important hormone for various reasons in our body as described in the article. Deficiency or excess of it is harmful. PTH's instigation of the PTH 1 receptor might help the organization of human fat tissue by coordinating lipolysis and thermogenesis. Plan of action is at point-of-care.

Patients with ongoing kidney infections showed higher intraoperative post-excision parathyroid chemical levels. Regardless, renal control didn't impact the ability to make intraoperative parathyroid texture over an extended timeframe, nor did renal end affect the credibility of gathering the information. In these ordinary people, intraoperative parathyroid substance on investigation stays obvious, yet additional periods might need to be seen as levels return to normal.
